# Identification of Compounds With Potential Dual Inhibitory Activity Against Drug Efflux Pumps in Resistant Cancer Cells and Bacteria: Protocol for a Systematic Review

**DOI:** 10.2196/66197

**Published:** 2025-06-05

**Authors:** Elina Beleva, Antonia Diukendjieva, Ilza Pajeva, Ivanka Tsakovska

**Affiliations:** 1 QSAR and Molecular Modelling Institute of Biophysics and Biomedical Engineering, Bulgarian Academy of Sciences Sofia Bulgaria; 2 Clinic of Hematology Military Medical Academy Sofia Bulgaria

**Keywords:** drug resistance, bacterial, neoplasm, reversing agents, efflux pump inhibitors, cancer cells, bacteria, protocols, systematic reviews, antimicrobial, PRISMA-P

## Abstract

**Background:**

Drug efflux mediated by transporter proteins is one of the major mechanisms conferring multidrug resistance (MDR) to antimicrobial agents in bacteria and to chemotherapeutics in cancer cells. Therefore, the development or identification of efflux modulators represents a promising strategy to overcome the resistant phenotype. Various chemical compounds have been tested in experimental studies as reversal agents either in combination with antimicrobial or anticancer drugs and have shown sensitizing activity in resistant bacteria or cancer cell lines. Owing to the common resistance mechanisms exhibited by bacteria and cancer cells, the identification of chemical agents with dual reversal activity offers a strategy to simultaneously combat antibacterial and cancer multidrug resistance.

**Objective:**

This study aims to conduct a systematic review to identify compounds that have shown activity in reversing antibacterial as well as cancer MDR mediated by drug efflux pumps and to summarize their structural and biological parameters responsible for the interactions with drug efflux pumps.

**Methods:**

The protocol has been developed in accordance with PRISMA-P (Preferred Reporting Items for Systematic Reviews and Meta-Analysis Protocols) guidelines. We searched PubMed and Scopus databases for abstracts of full-text peer-reviewed journal papers in English language published between January 2012 and September 2024. Only studies from in vitro experiments were considered if they used methods to detect changes in antibiotic sensitivity of resistant bacteria and chemosensitivity of resistant cancer cells upon treatment with efflux pump inhibitors. A total of 763 unique records were identified. Of them, 246 were selected for full-text review based on the eligibility criteria. Abstract screening was performed by 2 independent reviewers. As of March 1, 2025, the systematic review is at the stage of completed abstract screening. The next steps of the full-text review, study selection, data extraction, and risk of bias assessment will be performed by 2 independent reviewers as well. Main data elements will include a structural identifier of the tested inhibitor, bacterial strain, cancer cell line, methods proving reversal activity, half maximal inhibitory concentration, and other relevant quantitative estimates of reversal activity. Data synthesis will be performed as a narrative summary and the content will be curated in tabular and graphical form.

**Results:**

We anticipate that results from this study will outline the potential of various compounds to act as dual chemosensitizers and reverse both antimicrobial and cancer MDR.

**Conclusions:**

Our review will highlight the overlap between efflux pumps’ inhibition as a strategy to combat MDR in both bacterial and cancer cells and it will provide structured data for rational drug design of dual efflux pump inhibitors.

**Trial Registration:**

PROSPERO CRD42024548350; https://www.crd.york.ac.uk/PROSPERO/view/CRD42024548350

**International Registered Report Identifier (IRRID):**

PRR1-10.2196/66197

## Introduction

Drug resistance defined as the loss or diminution of pharmacological drug effects represents an important impediment to the success of medical treatments. Cancer and infection are the 2 medical contexts where the emergence of drug resistance is of utmost clinical importance as it may significantly compromise treatment outcomes. Both pathogenic microorganisms and human cancer cells have developed protective mechanisms to evade cytotoxicity triggered by pharmacological agents and one of the commonest multidrug resistance (MDR) mechanisms shared by both is the drug efflux mediated by transmembrane transport molecules [1].

Antimicrobial resistance (AMR) has been recognized by the World Health Organization (WHO) as a global public health threat and economic burden [2]. According to a report on the global burden of AMR, there were an estimated 4.95 million deaths worldwide associated with AMR in 2019 [3]. When compared with all underlying causes of death in the Global Burden of Disease 2019 AMR would have been the third leading cause of death after ischemic heart disease and stroke on the basis of the counterfactual scenario of no infection.

Cancer cells can also exhibit resistant phenotypes to the antineoplastic agents they have been exposed to in the course of anticancer drug treatment. Induced resistance to chemotherapy is responsible for nearly 90% of cancer deaths [4].

Drug efflux mediated by the overexpression of transport proteins recognizing and extruding a wide range of drugs, thus acting as efflux pumps, is one of the major mechanisms conferring resistance to antimicrobial agents in bacteria and to structurally unrelated chemotherapeutic agents in cancer cells (classical MDR phenotype) [5]. By extruding xenobiotics (cytotoxic agents and antibiotics) out of the cell, the efflux pumps decrease the intracellular concentration of drugs, thereby obviating their cytotoxic effects. Efflux pump inhibitors (EPIs), also called MDR modulators, are chemical compounds, either of natural or synthetic origin, that bind to the efflux pump and block the extrusion of other ligands, that is inhibit their efflux. In several studies, it has been demonstrated that the sensitivity of bacteria or cancer cells to antimicrobials or cytotoxic agents is reinstated when these are coadministered with an EPI. This ability to reverse MDR (MDR reversal) represents a possible strategy to overcome the MDR phenotype. Despite their differing origin, some bacterial and mammalian efflux pumps share the same or structurally similar ligands [6]. Owing to the common resistance mechanisms exhibited by bacteria and cancer cells, the development or identification of chemical agents that act as dual efflux pump inhibitors or modulators offers a promising avenue to tackle the problem of rising cancer and antimicrobial drug resistance.

In addition, a range of other applications might be considered. In some instances, dual inhibition is undesirable; thus, identifying dual inhibitors can prevent their misuse. For example, the inhibition of the *Staphylococcus aureus* efflux pump NorA, belonging to the Major Facilitator Superfamily (MFS) superfamily, by EPIs helps restore the effectiveness of antibiotics against resistant strains. However, many of these inhibitors also bind to P-glycoprotein, one of the most studied ABC transporters associated with MDR in cancer cells when overexpressed [7,8]. Under physiological conditions, P-glycoprotein inhibition is considered harmful as it can adversely affect the metabolism, distribution, and elimination of drugs. Therefore, identifying dual NorA and P-glycoprotein inhibitors can aid in characterizing selective NorA inhibitors [9]. Conversely, the identified inhibitors may be seen as bioavailability enhancers for P-glycoprotein substrate drugs proving beneficial for antibiotics with bioavailability challenges [10].

The primary objectives of this systematic review are (1) to gather information from recent scientific literature on compounds that have shown activity in reversing antimicrobial as well as cancer MDR mediated by drug efflux pumps, that is exhibit dual reversal activity; (2) to identify, systematize, analyze, and summarize their structural and biological parameters responsible for the inhibitory effect toward efflux pumps; and (3) to provide insights on potential mechanisms of interaction between EPI and drug efflux pumps.

## Methods

### Approach

The methodology of a systematic review has been implemented as it is suitable to identify in an exhaustive manner compounds from literature with evidence of dual reversing activity and to explore the intersection between antimicrobial and cancer MDR. The protocol has been developed in alignment with the PRISMA-P (Preferred Reporting Items for Systematic Reviews and Meta-Analysis Protocols) [11]. The PRISMA-P checklist can be found in Multimedia Appendix 1.

We have identified the research question within the Problem-Intervention-Comparator-Outcome (PICO) framework: what compounds that have been tested as efflux pump inhibitors have shown activity in reversing antimicrobial resistance in bacteria and multidrug resistance in cancer cells (Table 1) [12]?

In accordance with PRISMA-P, the protocol was registered in the PROSPERO (International Prospective Register for Systematic Reviews) on December 24, 2024 (registration number CRD42024548350).

**Table 1:** Concepts according to Population Intervention Comparator Outcome framework.

Population (P)	Bacteria resistant to antimicrobials and chemotherapy-resistant cancer cells
Intervention (I)	Efflux pump inhibitors and MDR^a^ modulators
Comparator (C)	No efflux pump inhibitors and MDR modulators
Outcome (O)	Reversal of resistance by binding to the efflux pump and reinstatement of antimicrobial sensitivity in bacteria and chemosensitivity in cancer cells

^a^MDR: multidrug resistance

### Ethical Considerations

Ethics approval was not applied due to the *in silico* nature of the study being a systematic review of already peer-reviewed and published studies according to the local institutional review board policy of Biomed.

### Eligibility Criteria

Eligibility criteria for the study selection are specified in Textbox 1. Studies are considered eligible if all inclusion and none of the exclusion criteria are met.

**Study characteristics to be used as criteria for eligibility for the review.**

**Inclusion criteria**

- Studies reporting data from in vitro experiments on multidrug-resistant bacteria or cancer cell lines.
- Studied populations are bacteria resistant to antimicrobials and chemotherapy-resistant cancer cells (including bacterial isolates from clinical specimens and cancer cells from resistant tumors).
- Studied resistance mechanism is the classical multidrug resistance phenotype, that is resistance induced by overexpression or activity of drug efflux pumps.
- Study design includes treatment of resistant bacteria or cancer cells with an efflux pump inhibitor (EPI) in the presence of antimicrobials or chemotherapy drugs.
- Used EPI is a chemical compound that interacts with the membrane transport protein.
- Reported outcome is the reversal of resistance, that is an increase in sensitivity towards antimicrobials or chemotherapeutics is detected in resistant bacteria or chemotherapy-resistant cancer cells upon the addition of an EPI to the treatment. As a measure of resistance reversal upon the addition of the EPI at least a half-maximal inhibitory concentration of the antibacterial or chemotherapy drug should be reported.
- Studies in the English language reporting original research data.

**Exclusion criteria**

- *In vivo* animal studies, clinical studies, or *in silico* studies without an *in vitro* validation experiment.
- Studies on populations other than that of interest: fungi, parasites, viruses, noncancerous cells or tissues, chemosensitive cancer cells or susceptible bacteria without a resistant counterpart, precancerous conditions, carcinoma in situ.
- Multidrug resistance phenotype is induced by other mechanisms, for example, mismatch repair system or alterations in the signal-transduction pathways.
- Study design does not use cotreatment with an EPI.
- Studies that test a reversal mechanism other than an EPI interacting with the transport protein, for example, inhibition of transport protein expression or inhibition of transporter activity by an autoantibody.
- Change in the sensitivity of resistant bacteria or cancer cells is not detected by a quantitative measure.
- Review articles, conference proceedings, posters, abstracts without full text, systematic reviews, doctoral theses, patents, preprints, and technical reports. Articles not in the English language.

### Data Sources

Published articles were collected from the electronic scientific abstract and citation databases PubMed and Scopus. We have decided on the use of the 2 databases as they provide wide coverage, are considered sources for reliable and reproducible experimental data, and provide optimal tools for electronic search of biomedical literature [13]. The search was time-limited to looking for papers published in the time span between January 1, 2012, and August 31, 2024. We have focused on studies published after 2011 because we identified a review by Amaral et al [5], published in 2012, that very comprehensively summarizes previous data on EPIs with activity on both bacterial and cancer cell efflux pumps.

### Search Strategy

The search strategy, exemplified but not exhaustive, is demonstrated in Textbox 2.

**Search strategies.**

**A. Search strategy for PubMed database**

1. (“Drug resistance” [tw] OR “Multidrug resistance” [tw] OR “Antimicrobial resistance” [tw] OR MDR [tw] OR AMR [tw] OR “Antibiotic drug resistance” [tw] OR “Drug-resistance” [tw] OR “Xenobiotic resistance” [tw] OR “Tumor multidrug resistance” [tw] OR “Tumour multidrug resistance” [tw]) AND (“Drug efflux pump*” [tw] OR

ABC [tw] OR “ATP-binding cassette transporter*” [tw] OR “P-glycoprotein-mediated drug resistance” [tw] OR “Efflux of antimicrobial agent*” [tw] OR “Efflux of anticancer agent*” [tw])

2. (“Drug resistance, multiple” [mh] OR “Drug resistance, bacterial” [mh] OR “Drug resistance, microbial” [mh] OR “Drug resistance, neoplasm” [mh] OR “Drug tolerance” [mh]) AND (“Multidrug resistance-associated protein*” [mh] OR “Multidrug resistance protein*” [mh] OR

“ATP-Binding Cassette Transporter*”)

3. #1 OR #2

4. “Multidrug resistance modulat*” [tw] OR “Multidrug-resistance revers* agent” [tw] OR Modulat* [tw] OR “Revers* multidrug resistance” [tw] OR Modifier [tw] OR Modulat* [tw] OR “Overcom* multidrug resistance” [tw] OR “Clinical modulat*” [tw] OR “Resistance-overcoming activity” [tw] OR Bind* [tw] OR Substrate [tw] OR Sensitize* [tw]

5. #3 AND #4

6. (Neoplas*[tw] OR cancer [tw] OR tumor [tw] OR tumour [tw] OR malignancy [tw]) AND (Microb* [tw] OR bact* [tw] OR microorganism* [tw])

7. Neoplasms [mh] AND (Bacteria [mh] OR “Bacterial infections” [mh])

8. #6 OR #7

9. #5 AND #8

10. Filters applied: from 2012/1/1 - 2024/8/31 to #9

11. Limitation: English language

**B. Search strategy for Scopus database**

( TITLE-ABS-KEY ( ( inhibit* OR substrate* OR bind* OR ligand OR modulat* ) AND ( efflux AND pump OR efflux AND transport* OR efflux OR p-glycoprotein OR nora ) AND ( bacter* OR antibacter* ) AND ( cancer* OR anticanc* OR carcin* OR tumor* OR antitumor* OR antitumour* OR tumour* OR mammal* ) ) AND PUBYEAR > 2011 AND PUBYEAR < 2025 AND NOT PUBDATETXT ( “September 2024” OR “October 2024” OR “November 2024” OR “December 2024” ) ) OR ( TITLE-ABS-KEY ( resistance AND ( “efflux pump*” OR abc-transporter* OR mfs OR p-glycoprotein OR nora ) AND ( inhibit* OR modulat* OR bind* OR substrate OR “efflux pump inhibitor*” OR “pump inhibitor*” ) AND ( cancer OR tumor ) AND bacteria* ) AND PUBYEAR > 2011 AND PUBYEAR < 2025 AND NOT PUBDATETXT ( “September 2024” OR “October 2024” OR “November 2024” OR “December 2024” ) ) AND ( LIMIT-TO ( DOCTYPE , “ar” ) ) AND ( LIMIT-TO ( LANGUAGE , “English” ) )

### Selection Process

After applying the search strategy retrieved records were independently screened by 2 reviewers (AD and EB) by title and abstract based on the eligibility criteria. The discrepancy in results from studies selection after this initial stage was resolved by discussion with supervisors (IT and IP). Studies that have been selected for full-text review will undergo a second round of independent review by AD and EB and disagreements, again, will be resolved by the supervisors. Reviewers are blinded to each other’s decisions during the review process. For organizing, screening, conflict resolution, extracting, and archiving the results of the systematic review, we use Systematic Review Data Repository Plus (SRDR+) [14].

### Outcomes for Which Data Were Sought

Outcomes of interest are broadly categorized in Textbox 3.

**Outcomes.**

- Overcoming multidrug resistance in bacteria and cancer:
- Compounds that reverse resistance to antimicrobials in bacteria;
- Compounds that reverse resistance to chemotherapeutics in cancer cells;
- Knowledge about the mechanism of interaction between efflux pump inhibitors and a drug efflux pump
- Exploring the intersection between antimicrobial and cancer multidrug resistance

### Data Management, Extraction, and Main Data Elements

The authors will review selected studies to extract parameters of interest. Data to be extracted include (1) study title; (2) study author; (3) year; (4) International Union of Pure and Applied Chemistry name of EPI, Chemical Abstracts Service number; (5) structural identifier of EPI: Simplified Molecular-Input Line-Entry System, International Chemical Identifier; (6) cancer cell line; (7) bacterial strain; (8) drug efflux pump subjected to inhibition; (9) methods for proving reversal activity of inhibitor; (10) IC_50_ (half-maximal inhibitory concentration); (11) measures of MDR, for example, MDR reversal ratio; (12) intracellular drug accumulation; (13) percentage cell survival; (14) affinity constant; (15) drug-induced apoptosis; (16) effect of adenosine triphosphatase activity; (17) methods used for in silico analysis; and (18) protein-ligand interactions. In the data extraction process, one reviewer will extract data and another one will check the extracted data. Disagreements between reviewers will be resolved by supervisors. In case a chemical identifier is missing, such will be generated provided a chemical structure is given in the source. Alternatively, and for any other ambiguities, authors will be contacted.

### Risk-of-Bias Assessment

For the assessment of risk of bias a domain-based tool-ToxRTool (toxicological data reliability assessment tool) developed to evaluate the reliability of toxicological data, will be used [15]. ToxRTool addresses 5 specific criteria groups relevant to toxicological and pharmacological properties of compounds tested in in vitro experiments: (1) test substance identification, (2) test system characterization, (3) study design description, (4) study results documentation, and (5) plausibility of study design and results. Application of the tool leads to the assignment of each item to one of the four reliability categories by Klimisch: “reliable without restriction,” “reliable with restriction,” “not reliable,” and “not assignable” [16]. Two reviewers will independently apply the tool for each included item, record the numerical result leading to a reliability category, and justify the evaluation. Any discrepancies will be resolved through discussion with the supervisors. For quality assessment across studies, a development of GRADE (Grading of Recommendations Assessment, Development, and Evaluation) for preclinical studies will be used [17].

### Data Synthesis

Data synthesis will be performed as a narrative summary. Qualitative and quantitative content will be analyzed and presented as tables and figures. Data will be organized around the compounds tested as EPI both in bacteria and cancer cells. No meta-analysis is being planned due to the essence of the review question.

## Results

**Figure 1:**
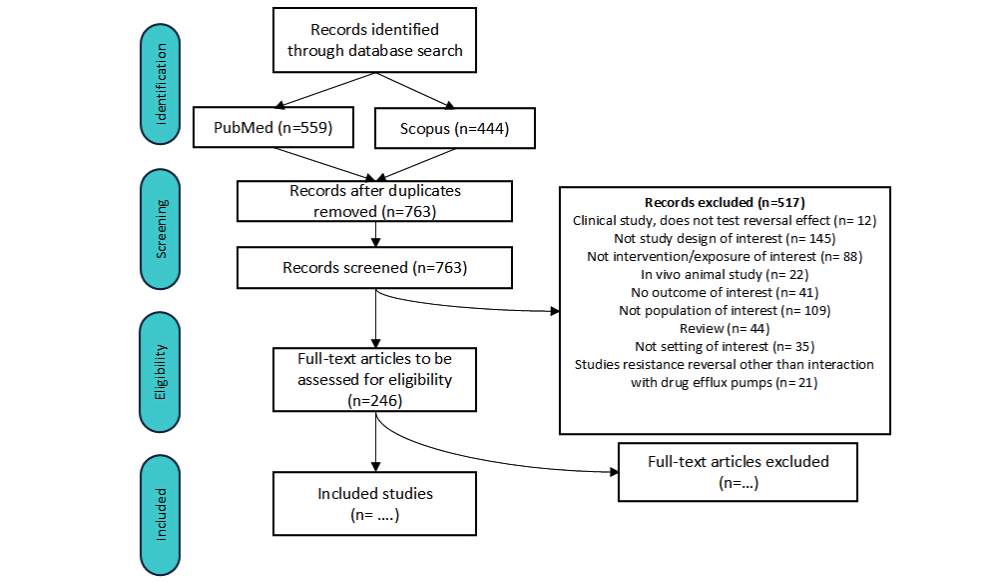
PRISMA flowchart of the selection and review process.

The protocol for this systematic review was developed in June 2024 and few pilot searches were performed. Initially time limitation was set between January 1, 2012, and June 30, 2024. Since there were differing views on the optimal search strategy, it took several rounds of discussion to reach a consensus. After a consensus was reached among the team members on the final combination of search strategies to use, the protocol was amended to expand the search up to September 2024. PubMed was searched on October 28, 2024, and Scopus was searched on February 12, 2025. Results from the searches were downloaded as csv files. A txt file containing a list with PMIDs from the PubMed search results and a csv file containing DOIs of the Scopus search results were fed into SRDR+ to fetch citations. SRDR+ was used to perform the abstract screening and to select records for further full-text screening. The results from the search and the abstract screening are presented in the PRISMA (Preferred Reporting Items for Systematic Reviews and Meta-Analyses) flowchart (Figure 1). In total, 1003 records were identified through database search. After the removal of duplicates, 763 unique records were screened by title and abstract by AD and EB. Each reviewer was blinded. Any screening conflicts that arose at this stage were resolved by the supervisors, IT and IP. During abstract screening, 517 records were excluded, and 246 records were accepted for the next step of full-text screening. Given the time requirements to perform full-text screening and data extraction, we envision this next step as compassable to execute by the end of August 2025. Data analysis will be performed by November 2025, and preparation of manuscript containing a report on the results from the systematic review and manuscript submission will be completed by February 2026.

## Discussion

### Overview

While efflux pumps of the ATP-binding cassette (ABC) superfamily are known to play a role in cancer resistance pathways, 6 bacterial efflux pump families have been identified. These include the ABC superfamily, as well as MFS, multidrug and toxic compound extrusion family, resistance nodulation cell division family, small multidrug resistance family, and proteobacterial antimicrobial compound efflux family [18,19]. Depending on the source of energy they use to pump out the substrates, the efflux pumps are categorized into primary transport proteins, which hydrolyze ATP for energy (ABC superfamily), and secondary transport proteins, which use the proton or ions gradient (proton motive force) as their energy source [20].

### Principal Findings

Exploration of the intersection between bacterial and cancer multidrug resistance has been identified as an interesting research topic since the findings that the extrusion of xenobiotic compounds by efflux pumps represents a shared resistance mechanism for both bacteria and cancer cells [1]. The proposed systematic review will critically analyze the scientific literature in the last 15 years to identify chemical compounds that have shown activity as EPIs in both resistant bacteria and resistant cancer cells. We expect that results from this study will demonstrate the potential of various compounds to act as dual chemosensitizers and reverse both antimicrobial and cancer multidrug resistance. The anticipated main finding of this systematic review would be the summary of data on chemical compounds with the ability to decrease resistance toward antimicrobial and antineoplastic agents. Our findings will be systematically summarized as a database to outline the available structural and biological parameters of the identified compounds relevant to their reversing activity. In addition, as another principal finding we expect to present an overview of the proposed mechanisms of interaction between drug efflux pumps and EPIs. Findings from in silico studies modeling the interactions between the small molecules and efflux pumps, when supported by in vitro studies, have not been systematically reviewed.

### Comparison With Previous Work

Even though numerous studies have evaluated chemical compounds of natural and synthetic origin as MDR reversal agents, in their majority they have focused either on resistance reversal in bacteria or in cancer [21-25]. Few studies have reported results from testing a compound simultaneously on bacteria and cancer cells in the same experimental setting [26-29]. Moreover, studies differ in the targeted efflux pump, in the population of interest (bacterial strain or type of cancer cells) as well as in the tested EPIs. Other reviews have already discussed compounds with the potential to dually inhibit efflux pumps in bacteria and cancer, albeit not in a systematic manner. However, some of those reviews focus on a single drug class and discuss efflux inhibition among a myriad of other cell-directed effects of the class [30,31]. Therefore, we felt the need to summarize available data on compounds with EPI activity from either separate or simultaneous experiments in bacteria and cancer cells along with aggregation of data on their structural and biochemical properties that might be effectively used for in silico drug design.

### Strengths

By attempting to summarize the results from experiments on various bacteria and cancer cells differing in terms of EPIs our review will highlight the overlap between efflux pump inhibition in bacterial and cancer cells as a strategy to combat MDR in both settings. Further, it will provide structured data for rational drug design of dual (bacterial and cancer) EPIs such as, but not limited to, pharmacophore generation, Quantitative Structure-Activity Relationship models, and molecular dynamics simulations.

### Limitations

The systematic review will have limitations. We envision heterogeneity between studies as the most significant limitation. We expect certain variability between studies with respect to the methodology used for proving reversal activity, experimental setting, bacterial strains, cell lines, and outcome measurements, all of which may render the comparison across studies difficult. There is also the risk of inherent reviewer bias which will be mitigated by independent blinded review and solicited input from experts on the subject. In addition, we will implement a standardized tool for assessing the risk of bias for each study. There is a chance that only a few compounds will be identified as dual ABC pump inhibitors after this review. Nevertheless, the review will screen the literature and provide comprehension of whether the topic of the intersection between bacterial and cancer MDR is plausible for exploration with further research endeavors.

### Future Directions

By providing curated data on dual EPIs we hope that this review will subsequently aid in the discovery of new compounds with EPI activity or guide the repurposing of already approved compounds into their clinical use as EPIs in conjunction with antibacterials or chemotherapeutics. Furthermore, the outcomes of this review may support the concept of using bacteria as a tool to screen and evaluate EPIs that could potentially be used to overcome MDR in cancer cells. Finally, the results from this review will generate perspectives for future research efforts in tackling drug resistance with potential implications in the clinical setting.

### Dissemination Plan

The findings of this peer review will be disseminated through a peer-reviewed journal article.

### Conclusion

This systematic review will provide a comprehensive analysis of EPIs that have been tested as reversal agents in resistant bacteria and cancer cells. Ultimately, it will identify those compounds that intersect as dual resistance sensitizers. This research will hopefully provide the basis for the development of protocols for in silico investigations of efflux pump inhibitors.

## Appendix

Multimedia Appendix 1 PRISMA-P checklist.

## Abbreviations

ABC: ATP-binding cassette
AMR: antimicrobial resistance
EPI: efflux pump inhibitor
GRADE: Grading of Recommendations Assessment, Development, and Evaluation
IC50: half-maximal inhibitory concentration
MDR: multidrug resistance
MFS: major facilitator superfamily
PICO: Problem Intervention Comparator Outcome
PRISMA: Preferred Reporting Items for Systematic Reviews and Meta-Analysis
PRISMA-P: Preferred Reporting Items for Systematic Reviews and Meta-Analysis-Protocols
PROSPERO: International Prospective Register for Systematic Reviews
SRDR+: Systematic Review Data Repository Plus
ToxRTool: toxicological data reliability assessment tool
WHO: World Health Organization

## Data Availability

The dataset generated or analyzed during this study will be made available in the SRDR+ repository.
